# ‘I wouldn’t get that feedback from anywhere else’: learning partnerships and the use of high school students as simulated patients to enhance medical students’ communication skills

**DOI:** 10.1186/s12909-015-0315-4

**Published:** 2015-03-07

**Authors:** Helen Cahill, Julia Coffey, Lena Sanci

**Affiliations:** 1 Youth Research Centre, University of Melbourne, Level 5, 100 Leicester St., 3010 Victoria, Australia; 2 Department of General Practice, The University of Melbourne, 200 Berkeley Street, Carlton, 3053 Victoria Australia

**Keywords:** Medical education, HEAADSSS, Young people’s perspectives, Medical students, Clinical skills, Education, Youth friendly

## Abstract

**Background:**

This article evaluates whether the use of high school students as simulated patients who provide formative feedback enhances the capacity of medical students in their fifth year of training to initiate screening conversations and communicate effectively with adolescents about sensitive health issues.

**Methods:**

Focus group interviews with medical students (n = 52) and school students aged 15–16 (n = 107) were conducted prior to and following involvement in *Learning Partnerships* workshops. Prior to workshops focus groups with school students asked about attitudes to help-seeking in relation to sensitive health issues, and following workshops asked whether the workshop had made a difference to their concerns. Prior to workshops focus groups with medical students asked about their needs in relation to initiating conversations with adolescents about sensitive health issues, and following workshops asked whether the workshop had made a difference to their concerns. Surveys were also completed by 164 medical students and 66 school students following the workshops. This survey featured 19 items asking participants to rank the usefulness of the workshops out of 10 (1 = not at all useful, 10 = extremely useful) across areas such as skills and understanding, value of learning activities and overall value of the workshop. SPSS software was used to obtain mean plus standard deviation scores for each item on the survey.

**Results:**

The *Learning Partnerships* workshops assisted medical students to improve their skills and confidence in communicating with adolescents about sensitive health issues such as mental health, sexual health and drug and alcohol use. They also assisted young people to perceive doctors as more likely potential sources for help.

**Conclusions:**

These findings suggest that the innovative methods included in *Learning Partnerships* may assist in broader education programs training doctors to be more effective helping agents and aid the promotion of adolescent friendly health care. This research provides evidence that a new way of teaching may contribute to enhancing doctors’ capacity and willingness to initiate screening conversations and enhance adolescents’ preparedness to seek help. This has implications for educational design, content and communication style within adolescent health.

**Electronic supplementary material:**

The online version of this article (doi:10.1186/s12909-015-0315-4) contains supplementary material, which is available to authorized users.

## Background

This article reports on research findings on the use of an innovative methodology in which high school students act as simulated patients and give client feedback to enhance the capacity of doctors to communicate effectively with adolescents about their health issues. The research aimed to identify the impact of this educational intervention related to the knowledge, skills and attitudes related to doctors’ communication for each of the participating parties.

Doctors complete their training with limited opportunities to advance the quality of their communication with the young people who will be their clients [1]. Family doctors are ideally placed to identify and respond to the common psychosocial burdens of young people, however, adolescents are reluctant to seek help for issues related to alcohol and other drug use, and sexual or mental health [2,3]. Opportunistic screening is a crucial strategy in addressing key adolescent health issues including suicide prevention [4,5]. Many health professionals feel they have insufficient training and skills to work effectively with adolescents [6-9]. A strong focus on adolescent health is needed within undergraduate and postgraduate health courses [6]. A recent review of studies on young people’s perspectives on health care found that the clinician’s attitude and demeanour, such as respect and friendliness and the quality of the clinician’s communication skills were crucial factors in adolescent-friendly health care [10]. Given the importance of these skills in providing effective health care for adolescents, and the reluctance of young people to be proactive help-seekers, it is crucial that health professionals receive adequate training in initiating screening communications with adolescents.

Simulated patients are increasingly used to train medical students and other health professionals [11]. This method provides experiential learning needed to master new skills [12,13] and is primarily used for communication training [14-17]. Rehearsal, formative feedback and performance assessment are key components of simulated patient exercises [18]. They can provide reliable and valid feedback on key competencies required to effectively conduct behaviour change consultations, including higher-order skills [12] such as communication, listening, engagement and goal setting [19]. Having the opportunity to rehearse and experiment with communication skills within the education space facilitates transfer of the skills to the doctor’s real world setting [11]. It is thus an important strategy in helping doctors to improve their practice [20]. This study adds to evidence supporting the use of simulated patients in medical students’ educational outcomes [11], and to calls for increased rigour in reporting research involving simulated patients [21].

*Learning Partnerships* aims to enhance medical students’ communication skills through participation in workshops with classes of high school students. Through these workshops, medical students are given the opportunity to practice their communication skills and receive formative feedback via simulated patient exercises with adolescents.

### Workshop programme

The *Learning Partnerships* education methodology was developed by the first author in 1993, initially for use in training adolescent fellows at the Centre for Adolescent Health. This was later incorporated into a study in the training of General Practitioners, conducted by Lena Sanci [22], and formed the basis of an education PhD in 2008 [4]*. Learning Partnerships* has been used in the University of Melbourne undergraduate medical curriculum in the Faculty of Medicine and the Centre for Adolescent Health, Royal Children’s Hospital since 2003.

Classes of medical students in the fifth year of their training participate in a two-hour workshop with classes of high school students (typically aged 15 – 16 years). Medical students participate in the workshop when on speciality rotations of women's health, child and adolescent health, aged care and mental health. They have yet to do their General Practice rotation in final year and have already done (in year 4) their general medicine and surgery terms in hospital settings. While they have learned the basic building blocks of communication in the earlier pre-clinical years (1–3) they have not yet applied these skills to children or young people in community settings, and have not yet learned how to engage with hidden agendas and health promotion through anticipatory guidance. During the period of research, seven medical education workshops were run, each with a separate class of medical students. Where possible the workshops take place in the school during scheduled class time and within a subject home (such as Drama or English). This places the activity within the school curriculum, thus contributing to its status and the sustainability of the program.

The medical students (in groups of 20–25) participate in one two-hour communication-training workshop with around 25 adolescent students. Each medical session is co-facilitated by a classroom teacher and a medical educator, using a detailed agenda to guide the process. The classroom teachers prepare the school students during their timetabled class time (about 3–5 lessons) assisting them to develop skills in authentic portrayal of the patient and techniques for providing formative feedback*.* Medical students attend one session, and groups of school students commonly participate in two sessions with different groups of medical students.

The medical students play the family physician and use the HEEADSSS psychosocial screening tool to practice asking questions about the simulated patient’s home, education, eating, activities, drug and alcohol use, sexual activity and safety [23,24]. The school students play the fictional character of “Jo”, which has been specially devised for this exercise (see Additional file 1). The adolescents participate both as actors playing the patient, and as coaches, providing feedback to the medical students on how well the communication is progressing in the simulation. The participants work in doctor-patient dyads for most of the workshop. The simulations are interrupted at each phase of the screening so as the school students can provide coaching and feedback to their partner. Additional coaching and demonstrations are provided in fishbowl mode to demonstrate key skills such as making the confidentiality statement, arranging for time alone with the adolescent patient, use of suitably framed normalising or non-judgemental smorgasbord questions, and explaining the purpose of the screening.

## Methods

We investigated the impact of the *Learning Partnerships* intervention on medical students’ personal and professional confidence in communicating with adolescents on wellbeing related issues. Ethics approval was obtained from the University of Melbourne (HREC 1237767.1). All participants received an information sheet describing the research and gave informed consent by signing a consent form prior to data collection. Written informed consent for participation in the study was also obtained from parent or guardian for participants under 18 years old.

A mixed-method approach using qualitative (focus groups) and quantitative (survey) methods was used to evaluate seven medical education *Learning Partnerships* workshops which ran between August and December 2012. Focus group interviews with medical students (n = 52) and school students aged 15–16 (n = 107) were conducted prior to and following involvement in *Learning Partnerships* workshops. Surveys were completed by 164 medical students and 66 school students following the workshops (see Additional files 2 and 3). The findings from the medical students and school students are discussed here (see Table 1). (For findings from other parties, and for more detail about the focus group questions see [25]).

**Table 1:** **Data collection**

	Data collection		
*Target group*	Pre-workshop focus group	Post-workshop focus group	Post-workshop survey
***Medical students***	2 focus groups (n = 13)	5 focus groups (n = 39)	n = 164
***School students***	7 focus groups (n = 38)	12 focus groups (n = 69)	n = 66

### Focus groups

Focus groups are semi-structured interview-like discussions with a small group of participants (4–12) [26]. The focus groups generated dynamic group discussion, and enabled participants to elaborate on, question or support the responses of other group members [27]. Focus groups had between 4–9 participants of mixed gender, and ranged from 15–30 minutes in length.

Two focus group interviews were conducted either immediately prior to or in the week prior to involvement in workshops with 13 medical students (5 females, 8 males) who were invited by researchers, and who volunteered to participate. Respondents were asked how they feel about initiating conversations with young people, and how confident they feel using the HEADSSS screening questions in consultations. A further five follow-up focus groups were conducted with 39 medical students (22 females, 17 males) immediately after the workshops. The respondents in the two pre-workshop focus groups also participated in the post-workshop focus groups. The post-intervention focus groups were used to explore what respondents perceived they had gained from the workshops and the degree to which this experience would inform their professional approach. Both pre-and post-interviews followed a topic guide.

Seven focus groups were also conducted with 38 school students (28 females, 10 males) prior to the workshops, and a further set of 12 focus groups were conducted post-workshop with 69 school students (47 females, 22 males). All focus groups were audio recorded and transcribed verbatim, and were analysed and coded thematically. Transcripts were coded separately by two researchers and compared to cross-check themes and ensure rigour [26,27]. An effort was made to include the same school students in both pre and post focus group interviews; however this was not always possible due to student absences. Pre-workshop focus groups were conducted with school students one to two weeks prior to participating in the first of two *Learning Partnerships* workshops. The number of focus groups and individual participants was higher in post- than pre-focus groups as focus groups were conducted opportunistically immediately following workshops whilst students were still present.

### Surveys

Paper surveys were distributed and completed immediately following each workshop by 164 medical students (95 females, 69 males, 10 did not answer) and 66 school students (39 females, 22 males, 5 did not answer). The one-page anonymous paper survey was completed by most of the 170 medical students who undertook the workshop (n = 164). This provides a snapshot of the attitudes of medical students to the workshop and supports the focus group data [28]. This survey featured 19 items asking participants to rank the usefulness of the workshops out of 10 (1 = not at all useful, 10 = extremely useful) across areas such as skills and understanding, value of learning activities and overall value of the workshop, and is presented as means with standard deviation. SPSS software was used to obtain mean plus standard deviation scores for each item on the survey. (For results see Tables 2 and 3).

**Table 2:** **Post-workshop survey findings with medical students**

**1. Skills and understandings**	**N**	**Mean**	**Std. deviation**
1.1 Understand importance of informing young people about confidentiality	164	8.41	1.465
1.2 Ability to communicate effectively about sensitive issues	164	8.34	1.428
1.3 Sense of purpose to contribute to the care of young people	164	7.72	1.857
1.4 Knowledge of how to apply the HEADSS psycho-social screening tool	164	8.41	1.490
1.5 Understand how to negotiate seeing adolescent without parent for part of the consultation	164	8.12	1.422
1.6 Understand the challenges adolescents can encounter in disclosing experiences relating to drugs or sex	164	8.38	1.433
1. 7. Understand importance of proactive screening with adolescent patients	164	7.91	1.659
**2. Activities useful for learning**			
2.1 Trying out techniques in role-play	164	8.58	1.527
2.2 Watching others role-play	163	7.93	1.843
2.3 The coaching and replay in fishbowl activity conducted by facilitator	163	7.71	1.898
2.4 Getting feedback and advice from the school students	163	8.52	1.561
2.5 Comments and feedback from peers	164	7.93	1.617
2.6 Comments and feedback from tutors	162	7.91	1.807
2.7 Use of the Hidden Thoughts technique to identify self-talk	150	6.51	2.379
2.8 Discussion following the activity	161	7.99	1.723
**3. Workshop outcomes overall**			
3.1.Increased confidence about the possibility of building positive relationships with adolescent patients	163	8.29	1.448
3.2 Provided better insight into the needs of adolescent patients	156	7.95	1.454
3.3 Provided opportunities to improve capacity to communicate well with adolescents	155	8.43	1.391

**Table 3:** **Post-workshop survey findings with school students**

**1. Skills and understandings**	**N**	**Mean**	**Std. deviation**
1.1 Learn about confidentiality at the doctors	66	8.70	1.525
1.2 Learn how to talk with doctors about sensitive issues	66	7.95	1.659
1.3 Talk with friends when they have problems with sex, drugs or mental health	66	6.86	2.190
1.4 Understand the doctor’s job in helping teenage patients with problems to do with sex, drugs or mental health	66	8.15	1.666
1.5 Develop your own confidence to talk about personal health problems	66	7.88	2.004
1.6 Feel more confident to talk to a doctor if needed	65	8.03	1.912
1.7 Feel more confident to help a friend to go to a doctor for advice on personal things like sex, drugs or mental health	66	7.56	2.120
1.8 Get a better understanding of problems or worries you have experience in the past	66	6.92	2.303
1.9 Get a better understanding of how to handle problems if they come up in the future	66	8.17	1.845
**2. Activities useful for learning**			
2.1Discussing the issues in the preparation workshops	66	7.38	1.795
2.2 Role-playing scenarios in the preparation workshops	66	7.91	1.795
2.3 Acting in the role-plays with the doctors	66	8.32	1.580
2.4 Watching the role-plays done with the doctors	65	7.85	2.188
2.5 The coaching and replay in fishbowl activity conducted by facilitator	66	8.03	1.839
2.6 Giving feedback and advice to the doctors	66	8.23	1.863
2.7 Listening to the comments and feedback from class mates	66	7.92	2.129
2.8 Listening to the comments from tutors and teachers	66	7.85	1.906
2.9 The Hidden Thoughts technique to unpack what people might be thinking	65	7.86	2.022
2.10 Discussion with the doctors	66	8.35	1.814
**3. Workshop outcomes overall**			
3.1 Increase your confidence in your own abilities to talk with adults	63	7.70	2.061
3.2 Increase your confidence that doctors may be useful when young people have personal health problems	63	8.13	1.782
3.3 Understand better how you might cope in the future if you get a doctor who is not good at talking with teenage patients	63	7.76	1.915
3.4 Increase your intention to encourage a friend to go to a doctor if they have a problem with sex, drugs or mental health	63	7.46	1.908
3.5 Make you more likely to go to a doctor if in the future you have a problem with sex, drugs or mental health	63	7.65	2.194

The following section discusses findings from the focus group interviews conducted prior to workshops which explored their concerns about communicating with adolescents about sensitive issues using the HEADSS screening tool.

## Results

### Medical students’ concerns about communicating with adolescents prior to workshops

Prior to workshops, medical students reported that they did not feel comfortable because of their own lack of practical experience in talking with adolescents, and because proactive screening necessitated asking about sensitive topics.

‘I guess I haven’t spoken to many adolescents, I don’t feel comfortable at this stage asking… or broaching those conversations’. (Medical student 1, male)

They expressed concerns about asking ‘intrusive’ questions. In this they appeared to believe that their duty was chiefly to be reactive (responding to the presenting problem) rather than proactive (conducting an opportunistic screening).

‘It depends on how important you think it is to bring it up, if it’s relevant to the presentation, how badly you need to know…if it’s not relevant to the symptoms they present, why would I ask about it?’ (Medical student 3, female)

Yeah, like ‘do I really need to know about this?’ It would seem strange to them, I think. Like, ‘why are they asking me this!’ (Medical student 6, female)

Many thought that it would be easier to initiate questions about mental health than sex because mental health would be something young people would ‘expect’ to discuss with their doctors:

‘If there wasn’t a presentation of symptoms, mental health would be a bit easier [than discussing sexual health]’. (Medical student 3, female)

‘I’d find it easier [asking about mental health], you can ask something like ‘have you been feeling down’. Asking that is easier’. (Medical student 1, male)

Some were concerned that initiating questions around sexual health might seem ‘a bit creepy’ unless there was a presenting complaint, and unless the patient understood why the questions were being asked, believing that it may be better to respond than to initiate.

Prior to workshops, young people in this study were asked what they thought the ‘most personal’ issue for young people would be out of sexual health, mental health or issues with drugs or alcohol (for a more detailed discussion of findings relating to young people in this study see Author 2013). Most of the school students said they thought mental health was the ‘most personal’ issue and that it would be more difficult to talk about than sexual health [29].

‘With mental health stuff, it’s really personal. And it’s not something you’d tell a doctor, it’s more something you’d tell friends…you wouldn’t trust them enough like you would a friend’. (School Student 2, year 9, male)

### Evaluating learning partnerships

Survey data shows that the medical students found the workshops valuable in improving their skills in communicating with young people and understandings of adolescent health needs. Respondents ranked the usefulness of the workshops out of 10 (1 = not at all useful, 10 = extremely useful) across areas such as skills and understanding, value of learning activities and overall value of the workshop. The mean was 7.72 or higher for all ‘skills and understanding’ items on the survey, with ‘Understand importance of informing young people about confidentiality’ (mean 8.41), ‘Knowledge of how to apply the HEADSS psycho-social screening tool’ (mean 8.41) and ‘Understand the challenges adolescents can encounter in disclosing experiences relating to drugs or sex’ (mean 8.38) as the mostly highly rated items. The highest rated learning activities were ‘Trying out techniques in role-play’ (mean 8.58), ‘Getting feedback and advice from the school students’ (mean 8.52) and ‘Discussion following the activity’ (mean 7.99). The items relating to the overall value of workshop were also highly rated. ‘Provided opportunities to improve capacity to communicate well with adolescents’ had a mean score of 8.43; ‘Increased confidence about the possibility of building positive relationships with adolescent patients’ scored a mean of 8.29, and ‘Provided better insight into the needs of adolescent patients’ scored a mean of 7.95. The survey data shows that the workshops were rated by medical students as highly useful in enhancing their skills and confidence initiating conversations with adolescents about sensitive issues, and for providing them with greater insight into the needs that young people had for adults to take the lead in initiating these conversations.

The focus groups conducted with medical students (n = 39) immediately after the workshops provided an opportunity to explore their experiences in greater depth. Respondents explained that they particularly valued the supportive and experimental learning space which provided opportunity to try out different communication strategies, and to re-play after receiving coaching.

‘It was useful learning to think on your feet. Learning different phrases, if they don’t work, ask again, try something else…you couldn’t do that with a real patient in a real situation’. (Medical student 17, female)

‘I thought it was great to see what all the different Jo [character] reactions were – it was great to figure out and practice how to respond to their emotions. Like, one shut down when I was asking about sex, but we were able to talk about other things for a while and then she opened up more’. (Medical student 1, male)

‘We got to have a go and make a mistake, it was ok to get it wrong because we were in such a supportive environment, to work out what works and what doesn’t (Medical student 5, female)

Medical students described that the immediacy and rapid feedback within the face-to-face interaction heightened their sense of accountability for their communication style as it became obvious that the patients would withhold information or ‘shut down’ if they did not ask suitably framed questions or if their manner was perceived as judgmental. The students gave emphatic feedback about the importance of non-judgmental style, use of proactive and educative questioning, as well as reassurances about confidentiality. The structure of authentic coaching was designed to heighten the medical students’ acceptance of the need to use a screening tool rather than to confine their attention to the presenting complaint, which in the case of this character was a spike in frequency and severity of asthma attacks following initiation into smoking.

It’s the subtleties that we learnt, little bits to learn how to be more sensitive about issues, and I think a lot of medical students needed those creases ironed out’. (Medical student 20, female)

‘The workshop helps you understand your own strengths and weaknesses and focus on ways of improving the overall experience for both parties involved. It will definitely help with real life situations!’ (Medical student 15, female)

The medical students valued the way in which the workshops provided a link between theory and practice. The practice helped to build their confidence in initiating and persisting with the screening conversation.

‘I feel a lot better actually. Getting the individual feedback from the students, as well as the actual practice – we do so much theory but we don’t ever get to put it into practice like that’. (Medical student 6, male)

‘It’s confidence building. I felt that getting experience will make me more confident’. (Medical student 8, male)

The medical students particularly valued the adolescent feedback provided to them following the role-plays. This provided an authentic, personalised and immediate feedback loop.

‘I liked the feedback from students. I got told ‘you probably need to smile more!’ I don’t think that’s something a patient would tell me, I wouldn’t get that feedback from anywhere else!’ (Medical student 14, male)

‘It’s helpful that they say ‘I would feel better if you asked it in this way’ or ‘if you’d said it like this, this would make me more comfortable’, but also the positive stuff like ‘you asking it this way was good’… it’s good to have that opportunity so that when we start GP practice we’ve been given some practice so you’re not thrown in there and like ‘this is the first adolescent I’ve talked to, how do I do this?” (Medical student 35, female)

‘Apart from these sorts of things [the workshop] there’s no other opportunity for feedback of this type’. (Medical student 38, female)

### School students

Survey data shows that the school students also found the workshops valuable for increasing their understanding and trust of doctors and their confidence that doctors could be a useful source of help on problems relating to sex, drugs and mental health. The mean for items in the ‘skills and understandings’ ranged from 6.92 to 8.70, with ‘Learn about confidentiality at the doctors’ (mean 8.70), ‘Get a better understanding of how to handle problems if they come up in the future’ (mean 8.17) and ‘Understand the doctor’s job in helping teenage patients with problems to do with sex, drugs or mental health’ (mean 8.15) as the top three highest rated items. The most highly rated learning activities for school students were ‘Discussion with the doctors’ (mean 8.35), ‘Giving feedback and advice to the doctors’ (mean 8.23) and ‘The coaching and replay in fishbowl activity conducted by facilitator’ (mean 8.03). The highest rated ‘overall outcome’ was the item ‘Increase your confidence that doctors may be useful when young people have personal health problems’ (mean 8.13).

In focus groups following the workshops, school students said the experience made doctors seem more ‘human’ and more approachable, and thus became more likely sources of help.

‘Now we know they are just there to help us; it’s not like they’re going to judge you’. (School Student 5, female)

‘Now you know how they learn it, so it’s not as intimidating’. (School Student 28, female)

‘They’re just as nervous as we are about talking about what’s wrong’. (School Student, 29, female)

Overall, the school students and medical students described that the encounters and activities in workshops gave them a greater understanding of each other, and more confidence in the possibility of relating effectively with each other.

## Discussion

It is known that medical students feel under-equipped to communicate with adolescents [30]. It is crucial to foster the skills that enable doctors to better respond to adolescents’ complex health needs [4,31,32]. Communication skills and friendliness create the sense of trust that is integral to youth-friendly health services [2,10]. This research suggests that using school students as simulated patients who also give feedback improves medical students’ confidence in communicating with and relating to adolescents. The approach used in *Learning Partnerships* program shares the same aspects known to be effective in standardised patient training techniques [11], but differs significantly through having school students act as both communication coaches and standardised patients. Research into this innovative education approach provides evidence that a new way of teaching may contribute simultaneously to enhancing the doctors’ confidence in their capacity to initiate screening conversations and enhancing the adolescents’ preparedness to seek help from doctors [33]. The data indicates that the workshops addressed medical students’ expressed need to develop their skills in conducting proactive helping conversations with young people, and that they addressed the reciprocal need that young people have to increase their own confidence in doctors as a viable source of help.

Both doctors and adolescents found that the workshops gave them a better insight into the challenges faced by the opposite party. The workshops provided medical students the opportunity to learn from adolescents’ feedback and comments. The face-to-face encounter helped medical students to become more confident communicating with adolescents about sensitive issues. In focus groups conducted prior to the workshops, school students identified that mental health was the ‘most personal’ issue, and that young people could feel too ‘intimidated’ to discuss personal issues such as this with a doctor. In the survey following workshops, school students identified they would be more likely to see doctors as sources of help on this matter. Other studies suggest that primary health providers often underestimate the difficulty young people have in discussing mental health and under-recognise mood disorders and suicidality among youth [5]. It is crucial therefore that doctors are educated about the sensitivity surrounding disclosures about mental health concerns and are equipped to address this proactively in their screening consultations with adolescents.

There is proof of concept for the potential of this educative technique to change the behaviour of established doctors. Research across a five-year period showed sustainability of change in doctors’ self-reported practices with adolescents [8,34]. A limitation of the current study is that due to practical issues such as class scheduling it was not possible to implement a strict pre- and post-test design. Further research is needed to identify whether medical students’ behaviour and communication skills change alongside their attitudes and increased confidence related to the experience of *Learning Partnerships*. Tracking medical students’ application of these skills in real practice, along with school students’ help-seeking and peer referral behaviours would add further evidence about the long-term value of the program.

## Conclusion

Implications can be drawn from this research into the use of innovative methods to assist in training doctors to be more effective helping agents. Implications for educational design include:

- use of authentic simulation;
- positioning the client as coach as well as simulated patient; and
- provision of an experimental learning space in which immediate formative feedback is provided together with the opportunity for rehearsal and re-play.

The implications for education content include:

- the importance of taking a proactive and opportunistic approach to initiating screening conversations with adolescents, and providing a tool to assist in this process;
- alerts to the sensitivity adolescents experience in disclosing their mental health concerns; and
- reiterations of the importance of explaining confidentiality to the adolescent patient.

The implications for communicative style include:

- the importance of explaining the reason for screening to the patient;
- use of non-judgemental questioning style; and
- selection of smorgasbord questions that name, guide and invite responses around areas of potential concern, rather than sole reliance on open-ended questions.

Further longitudinal research is required to further explore the impact of this education program; to investigate whether participating adolescents show increased rates of help-seeking or peer referral, and whether doctors when qualified go on to conduct opportunistic screening or initiate helping conversations with their adolescent patients.

## Additional files

Additional file 1:
**Patient Jo.**
Additional file 2:
**Medics Survey.**
Additional file 3:
**Student Survey.**
