# Supplementary material for: ‘I wouldn’t get that feedback from anywhere else’: learning partnerships and the use of high school students as simulated patients to enhance medical students’ communication skills
Source: BMC Med Educ. 2015 Mar 7;15:35. doi: 10.1186/s12909-015-0315-4 (PMC4355139; doi:10.1186/s12909-015-0315-4)
Supplement: Additional file 1: — Patient Jo. [file 12909_2015_315_MOESM1_ESM.docx]

**Additional file 1. Case character**

Patient Jo: developed for school students aged 15-16.

# Patient: Jo. Age 16. (Played as female or male, depending on sex of the actor).

Presenting issue:

Jo is brought to the doctor by mum. S/he has asthma, in the past under control, but recently a problem. Had a bad asthma attack at a party one Saturday night, and some subsequent smaller attacks. Friends didn’t realise what was happening at the party as they thought he/she was just drunk, but later called an ambulance. Jo has never told mum the reason for the serious attack which was a combination of smoking cigarettes and cannabis.

**Home:**

Jo lives with mum as only child. Sees dad every second weekend, but dad has new wife and 2 toddlers so Jo doesn’t go there so much. Jo’s mum is very protective about the asthma and used always to control the medications. Now Jo does– but there are often two to three days in the week when he/she forgets to take the preventer.

**School:**

Jo likes practical subjects like art and has average grades. School is boring but it is good to be there with friends. Jo does the minimum study required to get by at school.

**Activities:**

Favourite sport was basketball but had to give it up because Dad too busy with his new family, and Mum too busy to drive him/her to matches. Started going to parties last year. Nowadays goes to parties or gatherings are about every three weeks.

**Drugs:**

Jo started some experimental smoking last year. Now smokes around 4 cigarettes most days at school (before, at lunch and after school) but will smoke more at parties if cigarettes are available. Smoking does bring on asthma sometimes. Has tried cannabis a few times at parties. At the party when s/he had the bad asthma attack, this was after smoking a joint, drinking beer and smoking quite a few cigarettes. Also the room was very smoky. Jo’s Mum doesn’t know about the smoking, cannabis-use, or the use of alcohol. Jo hasn’t tried other drugs. Jo does not admit to mum that s/he is a smoker. Jo has been drunk about 5 or 6 times when drinking spirits, but usually just gets tipsy and usually drinks beer. Jo is reluctant to disclose any drug use unless the doctor is non-judgemental and gives reassurances about confidentiality.

**Sex:**

Two months ago at a party Jo got drunk and had accidently ended up having unprotected sex with someone who had a reputation for “getting around” a lot. Regretted and worried about this afterwards. This was the only time for Jo and no girlfriend or boyfriend at the moment. Deep down Jo is worried about sexually transmitted disease – could s/he have caught one and not know it? S/he would really like this question answered just to get it off his/her mind. Girl Jo –got her period the next Monday after that party so only had one day of worry about being pregnant. Jo will not disclose this story unless he/she feels safe to do so.

**Self-harm:**

Jo feels down sometimes, especially about Dad not being interested in him/her anymore and mum being such a nagging sort, but not so down that s/he thinks of hurting him/herself. Jo gets as low as 4 or 5 out of ten in mood sometimes, but most days is around a 6 to 7 out of ten (if ten is really happy and 1 is really down). In primary school Jo used to be happier (around 7 – 9) but that was before his/her parents split up.
