# Supplementary material for: ‘I wouldn’t get that feedback from anywhere else’: learning partnerships and the use of high school students as simulated patients to enhance medical students’ communication skills
Source: BMC Med Educ. 2015 Mar 7;15:35. doi: 10.1186/s12909-015-0315-4 (PMC4355139; doi:10.1186/s12909-015-0315-4)
Supplement: Additional file 2: — Medics Survey. [file 12909_2015_315_MOESM2_ESM.doc]

##### LEARNING PARTNERSHIPS PROJECT

##### MEDICAL STUDENTS EXIT SURVEY

##### Date: ______ Age: ____ Gender: M / F

| **A. Skills and understandings**: To what extent did participating in the workshop with the students enhance your: | | | | | | | | | | |
| --- | --- | --- | --- | --- | --- | --- | --- | --- | --- | --- |
| Scale from 1 = not at all useful--- *to 10 = extremely useful* | 1 | 2 | 3 | 4 | 5 | 6 | 7 | 8 | 9 | 10 |
| 1. Understanding of the importance of informing young people about confidentiality |  |  |  |  |  |  |  |  |  |  |
| 1. Ability to communicate effectively with adolescents about sensitive issues |  |  |  |  |  |  |  |  |  |  |
| 1. Sense of purpose or aspiration about contributing to the care of young people |  |  |  |  |  |  |  |  |  |  |
| 1. Knowledge of how to apply the HEADSS psycho-social screening tool |  |  |  |  |  |  |  |  |  |  |
| 1. Understanding how to negotiate to see the adolescent without parent for part of the consultation |  |  |  |  |  |  |  |  |  |  |
| 1. Understanding of the challenges adolescent patients can encounter in disclosing about experiences relating to drugs or sex |  |  |  |  |  |  |  |  |  |  |
| 1. Understanding of the importance of proactive screening with adolescent patients |  |  |  |  |  |  |  |  |  |  |

| **B. What helped you learn:** To what extend did the following activities help you learn | | | | | | | | | | |
| --- | --- | --- | --- | --- | --- | --- | --- | --- | --- | --- |
| *scale from 1 = not at all useful to 10 = extremely useful* | 1 | 2 | 3 | 4 | 5 | 6 | 7 | 8 | 9 | 10 |
| 1. Trying out techniques in role-play |  |  |  |  |  |  |  |  |  |  |
| 1. Watching others role-play |  |  |  |  |  |  |  |  |  |  |
| 1. The coaching and replay in fishbowl activity conducted by facilitator |  |  |  |  |  |  |  |  |  |  |
| 1. Getting feedback and advice from the school students |  |  |  |  |  |  |  |  |  |  |
| 1. Comments and feedback from peers |  |  |  |  |  |  |  |  |  |  |
| 1. Comments and feedback from tutors |  |  |  |  |  |  |  |  |  |  |
| 1. Use of the Hidden Thoughts technique to identify self-talk |  |  |  |  |  |  |  |  |  |  |
| 1. Discussion following the activity |  |  |  |  |  |  |  |  |  |  |

| **C. Overall learning:**  To what extent did participating in the workshop with the school students: | | | | | | | | | | |
| --- | --- | --- | --- | --- | --- | --- | --- | --- | --- | --- |
| *From 1 = very low to 10 = very high* | 1 | 2 | 3 | 4 | 5 | 6 | 7 | 8 | 9 | 10 |
| 1. increase your confidence about the possibility of building positive relationships with adolescent patients |  |  |  |  |  |  |  |  |  |  |
| 1. provide you with better insight into the needs of adolescent patients |  |  |  |  |  |  |  |  |  |  |
| 1. provide you with opportunities to improve your capacity to communicate well with adolescents |  |  |  |  |  |  |  |  |  |  |

| **D. Value of the workshop:** | | | | | | | | | | |
| --- | --- | --- | --- | --- | --- | --- | --- | --- | --- | --- |
| *From 1 = not at all useful to 10 = extremely useful* | 1 | 2 | 3 | 4 | 5 | 6 | 7 | 8 | 9 | 10 |
| What is the overall score you would give this workshop in terms of its value in contributing to your learning? |  |  |  |  |  |  |  |  |  |  |

### E. Your overall appraisal of the value of this activity for medical students

# Not useful

# Low value

# Moderately Useful

# Highly Useful

# Extremely Useful
