# Supplementary material for: ‘I wouldn’t get that feedback from anywhere else’: learning partnerships and the use of high school students as simulated patients to enhance medical students’ communication skills
Source: BMC Med Educ. 2015 Mar 7;15:35. doi: 10.1186/s12909-015-0315-4 (PMC4355139; doi:10.1186/s12909-015-0315-4)
Supplement: Additional file 3: — Student Survey. [file 12909_2015_315_MOESM3_ESM.doc]

##### LEARNING PARTNERSHIPS PROJECT

##### SCHOOL STUDENTS EXIT SURVEY (medical program)

##### Date: ______ Age: ____ Gender: M / F

| **A. Skills and understandings**: How useful did you find the workshops with the doctors in helping you to: | | | | | | | | | | |
| --- | --- | --- | --- | --- | --- | --- | --- | --- | --- | --- |
| *Scale from 1 = not at all useful--- to 10 = extremely useful* | 1 | 2 | 3 | 4 | 5 | 6 | 7 | 8 | 9 | 10 |
| 1. Learn about confidentiality at the doctors |  |  |  |  |  |  |  |  |  |  |
| 1. Learn how to talk with doctors about sensitive issues |  |  |  |  |  |  |  |  |  |  |
| 1. Talk with friends when they have problems with sex, drugs or mental health |  |  |  |  |  |  |  |  |  |  |
| 1. Understand the doctor’s job in helping teenage patients with problems to do with sex, drugs or mental health |  |  |  |  |  |  |  |  |  |  |
| 1. Develop your own confidence to talk about personal health problems |  |  |  |  |  |  |  |  |  |  |
| 1. Feel more confident to talk to a doctor if needed |  |  |  |  |  |  |  |  |  |  |
| 1. Feel more confident to help a friend to go to a doctor for advice on personal things like sex, drugs or mental health |  |  |  |  |  |  |  |  |  |  |
| 1. Get a better understanding of problems or worries you have experience in the past |  |  |  |  |  |  |  |  |  |  |
| 1. Get a better understanding of how to handle problems if they come up in the future |  |  |  |  |  |  |  |  |  |  |

| **B. What helped you learn:** To what extend did the following activities help you? | | | | | | | | | | |
| --- | --- | --- | --- | --- | --- | --- | --- | --- | --- | --- |
| *scale from 1 = not at all useful to 10 = extremely useful* | 1 | 2 | 3 | 4 | 5 | 6 | 7 | 8 | 9 | 10 |
| 1. Discussing the issues in the preparation workshops |  |  |  |  |  |  |  |  |  |  |
| 1. Role-playing scenarios in the preparation workshops |  |  |  |  |  |  |  |  |  |  |
| 1. Acting in the role-plays with the doctors |  |  |  |  |  |  |  |  |  |  |
| 1. Watching the role-plays done with the doctors |  |  |  |  |  |  |  |  |  |  |
| 1. The coaching and replay in fishbowl activity conducted by facilitator |  |  |  |  |  |  |  |  |  |  |
| 1. Giving feedback and advice to the doctors |  |  |  |  |  |  |  |  |  |  |
| 1. Listening to the comments and feedback from class mates |  |  |  |  |  |  |  |  |  |  |
| 1. Listening to the comments from tutors and teachers |  |  |  |  |  |  |  |  |  |  |
| 1. The Hidden Thoughts technique to unpack what people might be thinking |  |  |  |  |  |  |  |  |  |  |
| 1. Discussion with the doctors |  |  |  |  |  |  |  |  |  |  |

| **C. Overall learning:**  To what extent did participating in the workshops with the doctors: | | | | | | | | | | |
| --- | --- | --- | --- | --- | --- | --- | --- | --- | --- | --- |
| *From 1 = very low to 10 = very high* | 1 | 2 | 3 | 4 | 5 | 6 | 7 | 8 | 9 | 10 |
| 1. increase your confidence in your own abilities to talk with adults |  |  |  |  |  |  |  |  |  |  |
| 1. increase your confidence that doctors may be useful when young people have personal health problems |  |  |  |  |  |  |  |  |  |  |
| 1. Understand better how you might cope in the future if you get a doctor who is not good at talking with teenage patients |  |  |  |  |  |  |  |  |  |  |
| 1. Increase your intention to encourage a friend to go to a doctor if they have a problem with sex, drugs or mental health |  |  |  |  |  |  |  |  |  |  |
| 1. Make you more likely to go to a doctor if in the future you have a problem with sex, drugs or mental health |  |  |  |  |  |  |  |  |  |  |

| **D. Value of the workshop:** | | | | | | | | | | |
| --- | --- | --- | --- | --- | --- | --- | --- | --- | --- | --- |
| *From 1 = not at all useful to 10 = extremely useful* | 1 | 2 | 3 | 4 | 5 | 6 | 7 | 8 | 9 | 10 |
| What is the overall score you would give this workshop in terms of its value? |  |  |  |  |  |  |  |  |  |  |

**E. Your overall rating of the value of this activity for school students**

- Not useful
- Low value
- Moderately Useful
- Highly Useful
- Extremely Useful

##### LEARNING PARTNERSHIPS PROJECT

##### SCHOOL STUDENTS EXIT SURVEY (teacher education program)

##### Date: ______ Age: ____ Gender: M / F

| **A. Skills and understandings**: How useful did you find the workshops with the teachers in helping you to: | | | | | | | | | | |
| --- | --- | --- | --- | --- | --- | --- | --- | --- | --- | --- |
| Scale from 1 = not at all useful--- *to 10 = extremely useful* | 1 | 2 | 3 | 4 | 5 | 6 | 7 | 8 | 9 | 10 |
| 1. Have a better understanding about what it is like for teachers |  |  |  |  |  |  |  |  |  |  |
| 1. Understand how to ask a teacher for help if you need it |  |  |  |  |  |  |  |  |  |  |
| 1. Develop your own confidence to talk one to one with teachers |  |  |  |  |  |  |  |  |  |  |
| 1. Get a better understanding of how teachers and students can work well together |  |  |  |  |  |  |  |  |  |  |

| **B. What helped you learn:** To what extend did the following activities help you? | | | | | | | | | | |
| --- | --- | --- | --- | --- | --- | --- | --- | --- | --- | --- |
| *scale from 1 = not at all useful to 10 = extremely useful* | 1 | 2 | 3 | 4 | 5 | 6 | 7 | 8 | 9 | 10 |
| 1. Discussing issues in the preparation workshops |  |  |  |  |  |  |  |  |  |  |
| 1. Role-playing scenarios in the preparation workshops |  |  |  |  |  |  |  |  |  |  |
| 1. Acting in the role-plays with the teachers |  |  |  |  |  |  |  |  |  |  |
| 1. Watching the role-plays done with the teachers |  |  |  |  |  |  |  |  |  |  |
| 1. The coaching and replay in fishbowl activity conducted by facilitator |  |  |  |  |  |  |  |  |  |  |
| 1. Giving feedback and advice to the teachers |  |  |  |  |  |  |  |  |  |  |
| 1. Listening to the comments and feedback from class mates |  |  |  |  |  |  |  |  |  |  |
| 1. Listening to the comments from tutors and teachers |  |  |  |  |  |  |  |  |  |  |
| 1. The Hidden Thoughts technique to unpack what people might be thinking |  |  |  |  |  |  |  |  |  |  |
| 1. Discussion with the teachers |  |  |  |  |  |  |  |  |  |  |

| **C. Overall learning:**  To what extent did participating in the workshops with the teachers: | | | | | | | | | | |
| --- | --- | --- | --- | --- | --- | --- | --- | --- | --- | --- |
| *From 1 = very low to 10 = very high* | 1 | 2 | 3 | 4 | 5 | 6 | 7 | 8 | 9 | 10 |
| 1. increase your confidence in your own abilities to talk with adults |  |  |  |  |  |  |  |  |  |  |
| 1. increase your confidence that teachers may be useful when young people have personal health problems |  |  |  |  |  |  |  |  |  |  |
| 1. Increase your intention to encourage a friend to go to a teacher they like if they have a problem |  |  |  |  |  |  |  |  |  |  |
| 1. Make you more likely to go to ask a teacher you like for help if you have a problem |  |  |  |  |  |  |  |  |  |  |

| **D. Value of the workshop:** | | | | | | | | | | |
| --- | --- | --- | --- | --- | --- | --- | --- | --- | --- | --- |
| *From 1 = not at all useful to 10 = extremely useful* | 1 | 2 | 3 | 4 | 5 | 6 | 7 | 8 | 9 | 10 |
| What is the overall score you would give this workshop in terms of its value? |  |  |  |  |  |  |  |  |  |  |

**E. Your overall rating of the value of this activity for school students**

- Not useful
- Low value
- Moderately Useful
- Highly Useful
- Extremely Useful
